# Case-study: Patient with autism spectrum disorder and catatonia

**DOI:** 10.1192/j.eurpsy.2026.11073

**Published:** 2026-07-31

**Authors:** P. Terziivanova

**Affiliations:** 1 University Hospital for Neurology and Psychiatry “Sveti Naum”; 2 Medical University Sofia, Sofia, Bulgaria

## Abstract

**Introduction:**

Autism spectrum disorder (ASD) is a neurodevelopmental disorder with two key features – impaired social communication, and repetitive and restricted patterns of behaviours and interests. Catatonia has been recognized as a comorbid syndrome in 12-17 % of patients with ASD according to two prevalence studies. Autistic catatonia reflects psychomotor activity, speech, and responses to stimuli. ASD and Catatonia share a common pathophysiology - reduced GABA receptor functioning and altered D2/D3 receptor signaling in the basal ganglia.

**Objectives:**

A 23-year-old male with ASD is referred to an outpatient service due to changes in his psychomotor activity observed by his relatives. For the past four months, his movements had become slower, and he had difficulties in initiating and completing movements. His relatives described him as passive and “somehow more withdrawn than usual”.

**Methods:**

We used medical history, clinical observation, and Bush-Francis Catatonia Rating Scale (BFCRS), 23-item version.

**Results:**

All major developmental milestones were met. At the age of four, the patient communicated only with girls when the teachers were not in the room. He expressed symptoms of echolalia, repetitive speech, and selective mutism. When the patient was 10 years old, “his introversion and social withdrawal” became more significant. At the age of 11, he was clinically assessed with the Autistic Diagnostic Observation Schedule, Second Edition (ADOS-2), and his diagnosis was evaluated as ASD. He had a medical history of verbal hallucinations and aggressive behaviour at the age of 20. That condition was considered part of ASD and was treated with Risperidone for three weeks. The medical treatment with Risperidone was discontinued due to side effects – Parkinson like syndrome. At the age of 23, his movements slowed down, and his gait changed, resembling Parkinsonism. During the clinical interview, the patient spoke quietly, maintaining a mask-like facial expression. He resisted the clinician`s attempts to move his hands. The patient met clinical diagnostic criteria for Catatonia (BFCRS = 32). Patients’ relatives refused the suggested treatment with Lorazepam due to “its addictive potential”. Taking into consideration ASD, catatonia, and medical history for hallucinations, the second treatment option was an atypical antipsychotic. We started medical treatment with Cariprazine up to 3 mg daily. After one month of treatment, the severity of catatonic symptoms was reduced – BFCRS = 15. No side effects were reported.

**Conclusions:**

Cariprazine treatment reduced the severity of catatonic syndrome in the patient with ASD, described above. The observed improvement may reflect the mechanism of action of Cariprazine as a partial D2/D3 agonist. More data is needed because there are no randomized trials or larger observational studies validating the use of Cariprazine in Catatonia. Later development will be presented and discussed.

**Disclosure of Interest:**

None Declared

